# Structural basis for ligand-dependent dimerization of phenylalanine hydroxylase regulatory domain

**DOI:** 10.1038/srep23748

**Published:** 2016-04-06

**Authors:** Dipali Patel, Jolanta Kopec, Fiona Fitzpatrick, Thomas J. McCorvie, Wyatt W. Yue

**Affiliations:** 1 Structural Genomics Consortium, Nuffield Department of Clinical Medicine, University of Oxford, UK OX3 7DQ.

## Abstract

The multi-domain enzyme phenylalanine hydroxylase (PAH) catalyzes the hydroxylation of dietary I-phenylalanine (Phe) to I-tyrosine. Inherited mutations that result in PAH enzyme deficiency are the genetic cause of the autosomal recessive disorder phenylketonuria. Phe is the substrate for the PAH active site, but also an allosteric ligand that increases enzyme activity. Phe has been proposed to bind, in addition to the catalytic domain, a site at the PAH N-terminal regulatory domain (PAH-RD), to activate the enzyme via an unclear mechanism. Here we report the crystal structure of human PAH-RD bound with Phe at 1.8 Å resolution, revealing a homodimer of ACT folds with Phe bound at the dimer interface. This work delivers the structural evidence to support previous solution studies that a binding site exists in the RD for Phe, and that Phe binding results in dimerization of PAH-RD. Consistent with our structural observation, a disease-associated PAH mutant impaired in Phe binding disrupts the monomer:dimer equilibrium of PAH-RD. Our data therefore support an emerging model of PAH allosteric regulation, whereby Phe binds to PAH-RD and mediates the dimerization of regulatory modules that would bring about conformational changes to activate the enzyme.

Phenylalanine hydroxylase (PAH; EC 1.14.16.1) catalyzes the first step of l-phenylalanine (Phe) degradation via its hydroxylation to l-tyrosine, and serves to metabolize excess Phe in the diet. Inherited PAH deficiency, caused by mutations on the human *pah* gene, results in the autosomal recessive disorder phenylketonuria (PKU; OMIM 261600), which occurs at an average incidence of 1 in 10,000 live births^1^. PKU leads to mental retardation caused by elevated Phe levels in the blood and brain if it is not treated with a lifelong dietary restriction of Phe. To date more than 800 disease-causing *pah* mutations have been reported (http://www.biopku.org/pah)^2^, of which ~60% represent single amino acid, missense changes.

PAH, together with tyrosine hydroxylase (TH, EC 1.14.16.2) and tryptophan hydroxylase (TPH1 and TPH2 isozymes, EC 1.14.16.4), constitute the family of aromatic amino acid hydroxylases (AAAH) that are tetrahydropterin (BH_4_)-dependent, non-haem Fe(II) monooxygenase enzymes^3^. Like other AAAHs, PAH adopts a multi-domain architecture comprised of an N-terminal regulatory domain (RD, ~100 aa), a central catalytic domain (CD, ~300 aa) and a C-terminal multimerization helix (MH, ~40 aa) (Fig. 1A)^4^. Crystal structures of rat PAH lacking the C-terminal MH (RD + CD fragment)^5^, and of human PAH lacking the N-terminal RD (CD + MH fragment)^6^, have provided biochemical insight into the enzyme mechanism. The CD is responsible for the iron-mediated incorporation of one atom of molecular oxygen into the amino acid substrate and the reducing substrate BH_4_ to generate the hydroxylated products. The C-terminal MH mediates the formation of homo-tetramers that represent the native functional states^3^.

The role of the PAH-RD is less well defined. The enzymatic activity of PAH is known to be regulated at several levels^7^, including activation by substrate Phe^8^, inhibition by cofactor BH_4_, and also phosphorylation of Ser16^9^. Some, if not all, of these regulatory properties are likely mediated by the N-terminal RD, as deletion of the domain liberates the enzyme from activation and renders it constitutively active^10^,^11^. The inhibitory nature of the RD towards the CD is supported by the observation from the rat PAH structure of RD + CD that an N-terminal linker region of RD tails over to the catalytic domain and sterically blocks the active-site entrance^5^. While this manuscript was under review, the full-length structure of rat PAH in the absence of Phe has been reported^12^, revealing in molecular details the autoinhibitory nature of the N-terminal region. It has been suggested^13^,^14^ that the binding of Phe to the PAH active site or CD triggers a conformational change to the enzyme that leads to its activation. There is to date no structural data for ligand binding to the PAH-RD. PAH-RD contains an ACT (Aspartate kinase, Chorismate mutase and TyrA) fold commonly found in enzymes for binding small molecule ligands^15^. Indirect evidence for a Phe binding site within PAH-RD has been accumulated by site-directed mutagenesis^16^, gel filtration and HSQC NMR^17^. More recently, binding of Phe to PAH-RD has been linked to its ability to homo-dimerize^18^, and to increase the enzyme activity^19^. In this study, we have determined the high resolution crystal structure of human PAH-RD bound with Phe. Our data provide the structural evidence for a Phe binding pocket at the subunit-subunit interface of a PAH-RD dimer, and demonstrate that PAH-RD dimerization depends on Phe binding. Furthermore, we show that dimerization can be affected by a disease-associated Phe-binding mutation.

## Results

### PAH-RD has a binding site for Phe

**Figure 1:**
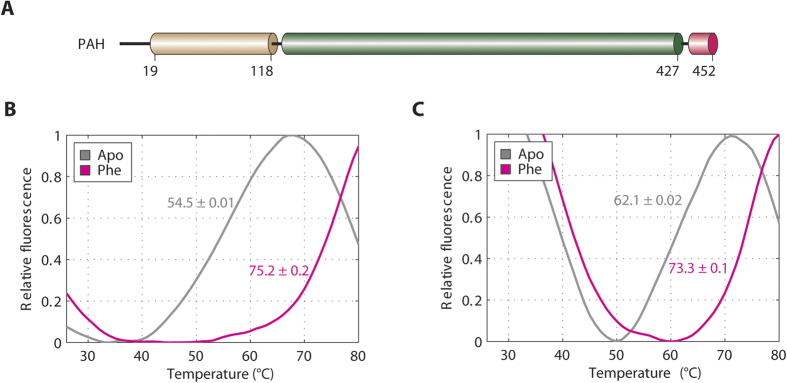
Interaction between PAH-RD and Phenylalanine. (**A**) Schematic of the regulatory domain (brown), catalytic domain (green) and tetramerization domain (pink) of the human PAH polypeptide. (**B**) DSF of the unliganded (grey line; Tm = 54.5 °C ± 0.01 SD) and Phe-bound (pink line; Tm = 75.2 °C ± 0.2 SD) hPAH-RD^1–118^. (**C**) DSF of the unliganded (grey line; Tm = 62.1 °C ± 0.02 SD) and Phe-bound (pink line; Tm = 73.3 °C ± 0.1 SD) hPAH-RD^19–118^.

The regulatory domain of PAH (PAH-RD) is located at the N-terminal 118 aa of the polypeptide (Fig. 1A). In the rat PAH structure of RD + CD^5^, aa 1–18 (same residue numbering as hPAH) was disordered, while aa 19–33 harbours an ‘autoregulatory motif’ for intramolecular active site gating^5^. To evaluate the binding of Phe to PAH-RD, we expressed hPAH-RD^1–118^ and hPAH-RD^19–118^ recombinantly in *E. coli*, and performed differential scanning fluorimetry (DSF). As-purified hPAH-RD^1–118^ exhibits a melting temperature (T_m_) of 54.5 °C, which was thermally stabilized to 75.2 °C in the presence of Phe, an indicator of ligand binding to the domain (Fig. 1B). hPAH-RD^19–118^ also shows a similar response to Phe (T_m_ shift from 62.1 °C to 73.3 °C) (Fig. 1C), suggesting that the N-terminal 18 aa segment is dispensable for Phe binding. Our solution data therefore support the notion of a Phe binding site in the PAH-RD, besides its known binding to the active site in the CD^20^.

### Structure of Phe-bound PAH-RD is a side-by-side homodimer

**Figure 2:**
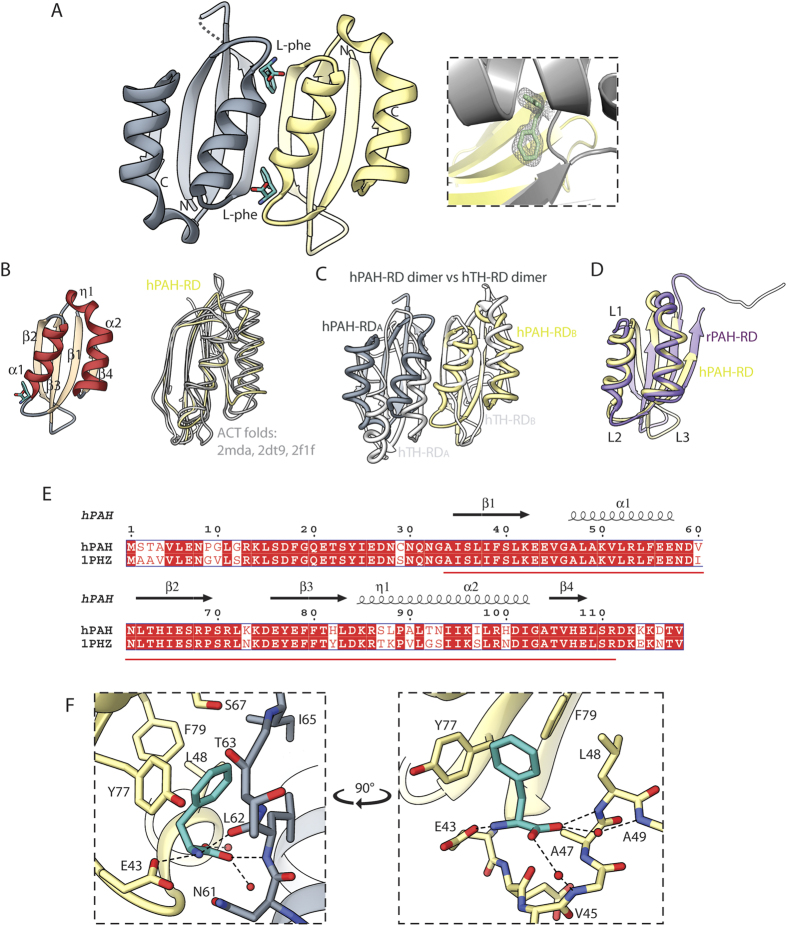
Crystal structure of hPAH-RD. (**A**) Ribbon representation of hPAH-RD dimeric structure, coloured grey and yellow for the two subunits. Phe is shown in sticks. (Inset) 2F_o_-F_c_ electron density map showing the bound Phe ligand. (**B**) *Left:* Topology of the ACT fold in hPAH-RD. *Right:* Structural superposition of hPAH-RD (yellow) with other ACT folds (grey) including PDB codes 2 mda, 2dt9 and 2f1f. (**C**) Structural superposition of hPAH-RD dimer (grey and yellow subunits) with hTH-RD dimer (white subunits). The two subunits of a dimer are annotated as A and B. (**D**) Structural superposition of hPAH-RD (yellow) with the rat counterpart (rPAH) extracted from its RD + CD structure (purple). (**E**) Alignment of the RD sequences between hPAH (this study) and rat PAH (PDB code 1 phz). Secondary structures from our hPAH-RD data are shown. Red line denotes the region covered in the hPAH-RD structure. (**F**) Phe binding site at the dimer interface of hPAH-RD^19–118^. The two subunits and Phe are colored as in *A*.

We next pursued a structural study of the Phe binding site of hPAH-RD. Following unsuccessful attempts to crystallize hPAH-RD^1–118^ and hPAH-RD^19–118^, we performed *in situ* limited proteolysis^21^, with the objective of removing flexible regions to facilitate crystal lattice packing. Trypsin-treated hPAH-RD^19–118^ yielded diffraction-quality crystals in the presence of Phe, allowing its structure determination at 1.8 Å resolution (Table 1). In the final model, the peptide regions Gly19-Gly33 and Asp112-Val118 are not present, which can be due to intrinsic disorder, or possible trypsinization, of the two regions. The structure of hPAH-RD is a homodimer (Fig. 2A), with two βαββαβ folds (one from each protomer; Fig. 2B) arranged side-by-side to form a continuous 8-stranded β-sheet, resulting in an internal 2-fold symmetry. The dimer interface is largely polar in nature, comprising main-chain/side-chain hydrogen bonds contributed from two amino acid regions Glu43-Leu48 and Asn61-Ser67, as well as from a bound Phe ligand (Supplementary Table 1). The PAH-RD side-by-side dimer closely resembles that of acetohydroxyacid synthase (AHAS) regulatory subunit (DALI Z-score 11.1, RMSD 1.6 Å, sequence identity 24%)^22^, but is topologically different from the regulatory domain of TH (4.9, 3.4 Å, 13%)^23^, another member of the AAAH family (Fig. 2C).

Omit Fo-Fc electron density map (Supplementary Fig. S1) revealed two Phe molecules bound to the hPAH-RD homodimer, at the interface of the two βαββαβ folds along the plane of the 2-fold axis (Fig. 2A, inset). Each Phe binding site (Fig. 2F) comprises residues from both protomers, including the ‘ExVxAL’ (aa 43–48) and ‘YxF’ (aa 77–79) sequence regions of one protomer, and the ‘NLTIxS’ (aa 61–67) region of the other. The residues involved in Phe-binding and dimer interaction are highly conserved among PAH orthologues, and also agree with a very recent NMR study on the Phe-binding region of rat PAH-RD^24^. To determine if Phe binding leads to conformational changes within the PAH-RD, we superimposed our Phe-bound hPAH-RD structure with that of unliganded PAH-RD, extracted from the rat PAH structure of RD + CD (83% sequence identity to hPAH; Fig. 2E). Significant Cα differences (RMSD >2 Å) between the two structures are found, and regions that undergo most conformational changes in the presence of Phe include (Fig. 2D): (i) loop L1 and the first turn of helix α1 (aa 42–49), which brings in residues of the ‘ExVxAL’ motif for Phe binding, and (ii) the segment (aa 59–75) containing loop L2, strand β2 and loop L3, which is displaced outwards to ‘open up’ the Phe-binding site.

### Phe-dependent dimerization of PAH-RD in solution

**Figure 3:**
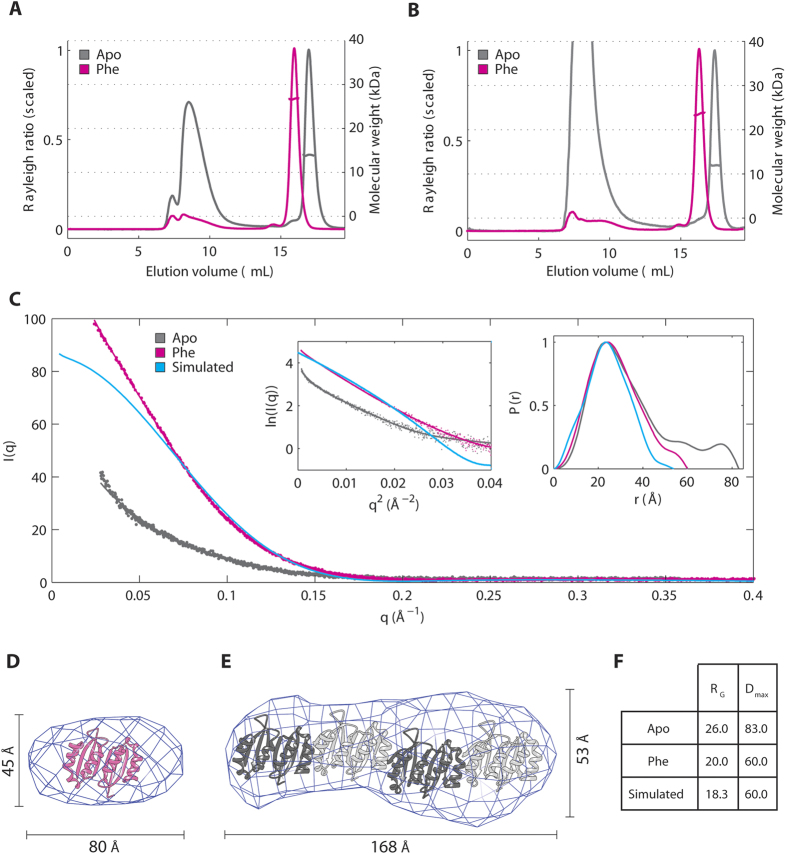
Phenylalanine stabilizes the dimeric conformation of PAH-RD. (**A**) SEC-MALS of unliganded (grey line) and Phe-bound (pink line) of hPAH-RD^1–118^. (**B**) SEC-MALS of unliganded (grey line) and Phe-bound (pink line) of hPAH-RD^19–118^. (**C**) SAXS profiles for hPAH-RD^19–118^ are plotted for the unliganded (grey line), Phe-bound (pink line) and crystal-structure-simulated (blue line, calculated using CRYSOL) data. Guinier plots (left) and real space P(r) distributions (right) are shown as inset. (**D**) *Ab initio* model of Phe-bound hPAH-RD^19–118^ derived from experimental SAXS data (using a *D*_max_ estimate of 60 Å), superimposed with the crystallographic dimer of hPAH-RD. (**E**) *Ab initio* model of unliganded hPAH-RD^19–118^ derived from experimental SAXS data (using a *D*_max_ estimate of 83 Å), revealing a rod-like shape as the dominant species, potentially accommodating four hPAH-RD crystallographic dimers as modelled. (**F**) R_G_ and D_max_ values calculated for the unliganded, Phe-bound and crystal-structure-simulated SAXS data of hPAH-RD.

To provide solution evidence for the crystallographic PAH-RD homodimer, the oligomeric state of hPAH-RD^1–118^ and hPAH-RD^19–118^ was evaluated by size exclusion chromatography with multi-angle light scattering (SEC-MALS). In the presence of Phe, both hPAH-RD proteins yielded an experimental MW (26.3 kDa and 22.4 kDa; theoretical monomer 13.5 kDa and 11.7 kDa respectively) consistent with a predominant dimer (Fig. 3A,B). A low-resolution *ab initio* envelope, generated from small-angle x-ray scattering (SAXS) measurement of hPAH-RD^19–118^ at multiple protein concentrations (to rule out concentration-dependent aggregation effect; see Experimental Procedure)(Fig. 3C), can accommodate one hPAH-RD dimer from the crystal structure (Fig. 3D). The larger volume for the *ab inito* model compared to our dimeric crystal structure is likely due to the tailing of the P(r) distribution curve (higher *D*_max_ region in Fig. 3C), and could be explained by (i) some aggregation present in the sample (also observed in the SEC-MALS data, Fig. 3B), and (ii) flexibility of the disordered N-terminal loop (aa 19–33) in solution. The latter is supported by a simulated P(r) distribution curve back-calculated from our PAH-RD structure encompassing aa 34–118 (Fig. 3C), which shows a smoother P(r) curve with a less pronounced tail region.

In the absence of Phe, both hPAH-RD^1–118^ and hPAH-RD^19–118^ proteins eluted as a mixed population of monomer (experimental MW 13.1 kDa and 11.3 kDa) and higher order aggregates, the latter indicated by the significant void volume peak at V_e_ = 8 ml (Fig. 3A,B). While a previous analytical ultracentrifugation study using the rat PAH-RD protein indicated a monomer-dimer equilibrium in the absence of Phe^18^, we did not observe any discernible peak for an *apo* dimer in SEC-MALS. The aggregated nature of unliganded PAH-RD is supported further from SAXS profiles (Fig. 3C), where the Guinier approximation and pairwise distribution function P(r) indicate an amyloid-like structure, with larger values for radius of gyration (*R*_*g*_) and maximal intraparticle dimension (*D*_max_) as compared to the Phe-present sample (Fig. 3F). Furthermore, *ab initio* modelling of unliganded PAH-RD^19–118^ (Fig. 3E) supports the presence of amyloid-like stacking which dominates the scattering signal, and is compatible with four PAH-RD dimers assembling with their β-sheets perpendicular to the long axis. Both SEC-MALS and SAXS data support the notion that in the absence of Phe, hPAH-RD is monomeric or prone to aggregation in solution, whereas the addition of Phe stabilizes the domain and reduces its aggregation tendency, likely through domain dimerization.

**Figure 4:**
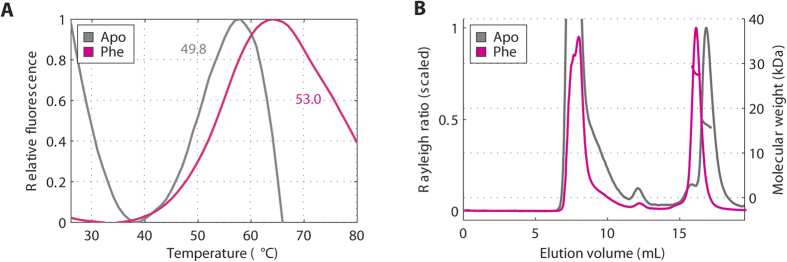
A phenylalanine binding mutation disrupts the propensity of hPAH-RD to homodimerize. (**A**) DSF of the unliganded (grey line; Tm = 49.8 °C ± 0.2 SD)) and Phe-bound (pink line; Tm = 53.0 °C ± 0.03 SD) hPAH-RD p.E76A. (**B**) SEC-MALS of unliganded (grey line) and Phe-bound (pink line) of PAH-RD p.E76A.

To verify that PAH-RD dimerization depends on its ability to bind Phe, we attempted to knock out Phe-binding residues by site-directed mutagenesis and study the oligomeric state of PAH mutants. To this end, we selected 4 disease causing mutations, namely p.G46S (c.136G > A), p.T63P_H64N (c.187_190delACCCinsCCCA), p.I65S (c.194T > G) and p.E76A (c.227A > C), based on their locations within or proximal to the Phe binding site revealed from the crystal structure. Three of them (p.G46S, p.T63P_H64N and p.I65S) result in insoluble protein when overexpressed in *E. coli*, highlighting their inherent destabilization. The remaining mutant p.E76A was soluble, and the purified protein is significantly compromised in its stabilisation by Phe, as reflected by a much reduced Tm shift in DSF upon the addition of 1 mM Phe (from 49.8 to 53.0 °C, ∆Tm = 3.2 °C), relative to its WT equivalent hPAH-RD^1–118^ (∆Tm = 20.7 °C) (Fig. 4A). SEC-MALS analysis shows that in the presence of Phe, p.E76A is not predominantly dimeric (as opposed to hPAH-RD^1–118^ and hPAH-RD^19–118^), but instead exists as a monomer-dimer equilibrium as illustrated by the overlap seen in the monomer/dimer peaks – a feature not present in the WT proteins. Furthermore, p.E76A also shows high-order aggregates in both Phe-present and Phe-absent samples (Fig. 4B), indicating its higher tendency for aggregation compared to wild-type, likely due to an insufficient capacity to bind Phe. Our data therefore illustrate a correlation between PAH-RD dimerization and its structural stability as well as ability to bind Phe.

### Dimerization of other AAAH regulatory domains does not depend on ligand binding

Among the AAAH family of enzymes, only PAH is shown to be allosterically activated by its own amino acid substrate, while the others (TH, TPH1, TPH2) are not, despite the presence of a regulatory domain in their N-terminal regions^4^ (Supplementary Fig. S2A). The NMR structure of the RD of human TH further shows an ACT-fold homodimer in the unliganded form^23^. We therefore isolated the RDs of human TH and TPH1 by recombinant expression, and explored if their oligomeric status is influenced by amino acid binding. In DSF, hTH-RD and hTPH1-RD are not thermally stabilized by l-Tyrosine and l-Tryptophan respectively, suggesting that they do not bind their cognate amino acid substrate (Supplementary Fig. S2B). Furthermore, SEC-MALS analysis of hTH-RD reveals a homodimer even in the absence of amino acid ligand (Supplementary Fig. S2C), confirming that its dimerization is not a consequence of l-Tyrosine binding.

## Discussion

It has long been suggested that phenylalanine serves as a homotropic ligand to phenylalanine hydroxylase, *i.e.* both as a substrate to the catabolic enzyme and as a regulator to its hydroxylation activity. The homotropic effect could be explained by Phe binding to one single site/region for both catalytic and regulatory functions, as proposed in some studies^13^,^14^, or by Phe binding to two distinct sites as proposed by others^8^. Much progress has since been made on biochemical and structural grounds to delineate the multi-domain organisation of PAH^3^, revealing the rationale for an N-terminal regulatory domain in gating substrate access to the C-terminal catalytic core^5^,^12^, and thereby modulating its enzyme activity. Recent solution studies have supported such an allosteric mechanism of PAH, involving a Phe-stabilized ACT fold during enzyme activation^18^,^19^,^24^. This study presents the structural evidence for Phe binding to PAH-RD as a ligand of the ACT fold, and provides the first high-resolution atomic view for such an allosteric binding site.

The key finding of our study is that the binding of Phe to PAH-RD restructures the ACT fold, forming the necessary inter-subunit dimer interface and the resultant Phe allosteric site. The Phe-bound hPAH-RD structure reported here is in agreement with examples of ACT-containing metabolic enzymes^25^,^26^, where their ACT folds function in concert, as dimers or higher-order oligomers stabilized by the binding of their cognate regulatory ligand (often an amino acid, as in PAH)^15^. To reconcile the difference with the recently reported rat PAH full-length structure, which did not reveal ACT:ACT interactions in the unliganded form^12^, we propose that the rat full-length PAH structure in the absence of Phe and our human PAH-RD structure in the presence of Phe could represent the unliganded ‘active-site blocking mode’ and the Phe-bound ‘multimerization mode’ of the domain, respectively. The two conformations therefore could exist in an equilibrium driven by the allosteric Phe ligand. Disturbed allostery is a key observation of PKU-associated mutations found in all three domains of PAH^27^, and our data gives the first structural viewpoint for this phenomenon.

**Figure 5:**
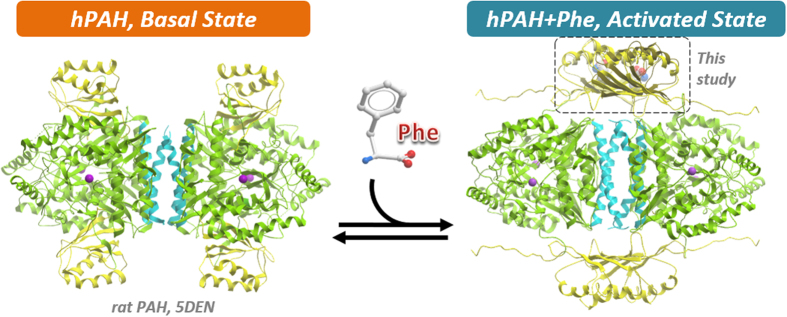
Proposed model of PAH activation by Phenylalanine. In the basal, unliganded state, as represented by the full-length rat PAH structure (PDB code 5DEN), the four RDs of a PAH tetramer do not interact with each other. Each RD (yellow) interacts with its corresponding CD domain (green) via the N-terminal loop region, resulting in steric hindrance of the CD active site. Phe binding relieves the steric hindrance due to homodimerization of RD, supported in this study, hence allowing unhindered access of substrates into the active sites.

It is therefore of no surprise that our PAH-RD dimer neatly confirms the proposed ACT interface that is integral to an active PAH tetramer model^28^ (Fig. 5). In this model, Jaffe *et al.* posited that a 90° rotation would be required to translate the PAH-RD from the active-site blocking mode into the multimerization mode, as a necessary path for the conversion of low-activity PAH dimers/tetramers into high-activity PAH tetramers. Such rotation of the PAH-RD would likely sequester the N-terminal auto-regulatory motif away from its inhibitory interaction at the active site^12^. Indeed there is precedence for long-range conformational movement in other ACT-domain enzymes, such as the DAH7PS enzyme where a ~110° reorientation of its RD, driven by Tyr binding, is part of the allosteric gating mechanism^26^,^29^. The inter-domain plasticity of PAH has also been supported by indirect solution studies, including limited proteolysis^30^, hydrogen/deuterium exchange^31^ and more recently, SAXS^12^. Additionally, the proposed model, coupled with our structure, explains the increase in volume^32^ and shift towards tetramers^33^ observed in the presence of Phe, making it highly likely that such a conformational change does occur in full-length PAH, as part of an allosteric activation mechanism in response to Phe binding. We are cognizant that our study involves an isolated PAH-RD that may not be fully extrapolated to its behaviour in the entire protein, therefore further biophysical and structural studies in the context of the full-length PAH tetramer are warranted to decipher the activating mechanism and underlying conformational changes of the PAH multi-domain enzyme.

Another notable finding is the observation that the regulatory domain of other AAAH enzymes (e.g. tyrosine hydroxylase and tryptophan hydroxylase) can also form the structural homodimer, albeit without the need to bind the cognate amino acid substrate. This corroborates with the NMR structure of TH-RD^23^ revealing an unliganded ACT dimer evolved with a distinct, ‘staggered’ interface that renders the canonical small molecule binding site no longer present. We posit that dimerization is a common mechanism for the ACT fold, which could be facilitated for some enzymes by ligand binding, but for others by alternative, post-translational triggers that remain poorly understood. It is also possible that the dependence (or not) of the ACT domain towards binding its cognate substrate is dictated by structural adaptation of the ACT dimer for either the ‘staggered’ (non-binding) or ‘side-by-side’ (binding) dimer interface. Structural characterization of the TPH-RD, not shown to respond to amino acid ligands, would help validate the hypothesis.

Hundreds of *pah* inherited mutations are the molecular cause of PKU, the most common inborn error of amino acid metabolism. PKU is often referred to as a conformational disorder^34^,^35^, in which protein destabilization, misfolding and aggregation are molecular hallmarks associated with disease^36^,^37^,^38^,^39^. Our solution data demonstrate the tendency of the N-terminal PAH-RD to form higher-order amyloid-like aggregates in the unliganded state, a phenomenon shown to be ameliorated by Phe stabilization *in vitro*. This supports previous claims that misfolding-induced aggregation is implicated in the *in vivo* disease mechanism^34^. A novel therapeutic approach, involving the use of small molecule target-specific ‘pharmacological chaperones’ (PCs) to stabilize aggregation-prone mutant proteins, is emerging as potential treatment for conformational disorders^35^,^40^. Relevant to this, PAH constitutes one of several multi-domain metabolic enzymes where a native regulatory ligand exists to activate the target via an allosteric site. The natural cofactor BH_4_ currently serves as a PC therapy for PKU, though it is not successful in treating all PKU disease alleles. Our study provides a rationale for the structure-guided drug design of PAH, using the PAH-RD as a target for small molecule activation. Rather than developing a PC molecule directed to the PAH active site, a small molecule that mimics Phe in stabilising the PAH-RD dimerization interface could activate the mutant enzyme above the threshold sufficient for relieving disease phenotypes, and prove useful as an alternative PC therapy, especially for those PKU mutations that are not BH_4_ responsive. The recent report of Phe-like molecules that bind PAH could potentially be acting in such a manner^41^, although caution will be needed to avoid cross-reactivity with the active site. Lessons could therefore be learnt from this and other examples of metabolic enzymes (e.g. cystathionine β-synthase, the cause of homocystinuria^42^; porphobilinogen synthase, the cause of ALAD porphyria^43^), for the design of novel PC molecules that target the allosteric domains as a stabilization strategy.

## Methods

### Expression and Purification of PAH, TH and TPH regulatory domains

A DNA fragment encoding the PAH regulatory domain (residues 1–118 or 19–118; GenBank number 4557819) was subcloned into the pNIC28-Bsa4 (GenBank Accession No. EF198106) vector incorporating an N-terminal TEV-cleavable His_6_-tag. The resulting plasmid was transformed into *E. coli* BL21(DE3), cultured in Terrific Broth at 37 °C until OD_600_ ~1.5, and induced with 0.5 mM IPTG for overnight growth at 18 °C. Cells were harvested and homogenized in buffer A (50 mM HEPES pH 7.5, 500 mM NaCl, 5 mM imidazole, 0.5 mM TCEP, EDTA-free protease inhibitor). Insoluble debris was removed by further centrifugation. Proteins were purified by passing cell extracts through a 1 ml HisTrap column pre-equilibrated with buffer A, and eluted with buffer B (buffer A + 250 mM Imidazole). Eluted fractions were treated with TEV protease at a protein:protease ratio of 1:20, and incubated overnight at 4 °C in order to cleave off the His_6_-tag. The tag-cleaved protein was applied onto a 1 ml HisTrap column, pre-equilibrated with GF buffer (50 mM HEPES, pH 7.5, 300 mM NaCl, 5% glycerol and 0.5 mM TCEP). The flow-through sample was applied onto a HiLoad 16/60 Superdex 75 column pre-equilibrated with GF buffer. The regulatory domains of human TH (residues 65–160; GenBank number 88900503) and TPH1 (residues 1–100; GenBank number 4759248) were subcloned into the pFB-Lic-Bse vector incorporating an N-terminal TEV-cleavable His_6_-tag, overexpressed in insect sf9 cells, and purified as above.

### Crystallization and structure determination of PAH-RD

Purified hPAH-RD was concentrated to 13.5 mg/ml, where l-Phenylalanine (Phe) was added to a final concentration of 10 mM. The sample was treated with trypsin at a protein:trypsin mass ratio of 1:100 immediately prior to crystallization set up. Crystals were grown by vapour diffusion in sitting drops at 20 °C. A sitting drop consisting of 100 nl trypsin-treated protein and 200 nl well solution was equilibrated against well solution containing 25% PEG 3350, 0.20 M NaCl and 0.1 M BIS-Tris pH 5.5. The crystals were mounted directly from the drop using 25% ethylene glycol as a cryoprotectant and flash-cooled in liquid nitrogen. Diffraction data was collected at the Diamond Light Source and processed with the CCP4 program suite^44^. The structure of hPAH-RD was solved by molecular replacement using the program PHASER^45^ with the *Chlorobium tepidum* prephenate dehydratase structure (PDB code 2QMX) as search model. Iterative cycles of restrained refinement and manual model building were performed using COOT^46^ and PHENIX^47^.

### Differential scanning fluorimetry

hPAH-RD was assayed for shifts in melting temperature caused by the presence of Phe in a 96-well PCR plate using an Mx3005p RT-PCR machine (Stratagene). Each reaction well (20 μl) consists of protein (10 μM in a buffer containing 50 mM Hepes, pH 7.5, 50 mM NaCl), SYPRO-Orange (Invitrogen) diluted 1,000×, and 1 mM Phe. Fluorescence intensities were measured from 25 °C to 96 °C with a ramp rate of 1 °C/min, as described^48^. The temperature shifts, Δ*T*_m_^obs^, for each ligand were determined^48^. The experiment was repeated three times and an error was calculated based on standard deviation.

### Small Angle X-ray Scattering

The hPAH-RD sample was purified by size-exclusion chromatography prior to sample preparation to remove any large aggregate species. hPAH-RD was prepared at 10 mg/ml with a final Phe concentration of 10 mM for the Phe-bound sample. Data collection was performed in batch mode, where samples were subjected to SAXS by flowing sample through an in-vacuum quartz capillary of 1.6 mm diameter. Data were collected using a Pilatus2M detector (Dectris, CH) at a sample-detector distance of 3914 mm and a wavelength of λ = 1 Å. The range of momentum transfer of 0.1 < *s* < 5 nm^−1^ was covered (*s* = 4πsinθ/λ, where θ is the scattering angle). Comparison of eighteen 10 s exposures was performed. Radiation damage was monitored for the batch mode by monitoring changes in radius of gyration in each frame, and no significant changes were observed. Samples were measured at three concentrations of 2.5 mg/ml, 5 mg/ml and 10 mg/ml for both unligand and liganded samples, to eliminate any concentration dependent aggregation during the SAXS experiment. The raw scattering curves for all concentrations were identical in profile, albeit poorer in the signal-to-noise ratio upon decreasing protein concentrations. The data were radially averaged and the scattering of the buffer was subtracted. The forward scattering *I*(0), radius of gyration *R*_g_, pair distribution of the particle *p*(r), and maximum dimension *D*_max_ were analyzed using ScAtter and the ATSAS suite of programs^49^.

### Size Exclusion Chromatography – Multiple Angle Light Scattering

SEC-MALS analysis was performed at 20 °C using an analytical Superdex 75 column (GE Healthcare) and a Shimadzu (Kyoto, Japan) chromatography system, connected in-line to a Heleos8 + multi-angle light scattering detector and an Optilab T-rEX refractive index (RI) detector (Wyatt Technologies, Goleta, CA). hPAH-RD was used at a final concentration of 3 mg/ml and Phe was used at a final concentration of 10 mM, in a buffer containing 10 mM Tris–HCl, pH 7.5, 300 mM NaCl, 2 mM β-mercaptoethanol. Samples measured with Phe were used in a buffer containing 10 mM Tris–HCl, pH 7.5, 300 mM NaCl, 2 mM β-mercaptoethanol, 10 mM Phe. Samples were injected in this system, and the resulting MALS, RI and UV traces processed in ASTRA 6 (Wyatt Technologies). On-column protein concentration was calculated from the differential RI, assuming dn/dc of 0.1850 ml/g. SEC-MALS data were fit with a two-state model to derive association parameters.

## Additional Information

**How to cite this article**: Patel, D. *et al.* Structural basis for ligand-dependent dimerization of phenylalanine hydroxylase regulatory domain. *Sci. Rep.* **6**, 23748; doi: 10.1038/srep23748 (2016).

## Supplementary Material

Supplementary Information

## Figures and Tables

**Table 1:** Summary of data collection and refinement statistics for the hPAH-RD structure.

Data Collection	
Space Group	P1
Wavelength (Å)	0.97623
a, b, c (Å)	36.14 37.41 64.08
α, β, γ (°)	98.94 103.83 94.79
Resolution range (Å)	34.81–1.80 (1.80–1.85)
Number of unique reflections*	28770 (2095)
R_sym_ (%)*	5.6 (50.2)
<I>/<σI>*	12.0 (2.1)
Completeness (%)*	91.1 (90.5)
Multiplicity*	1.7 (1.6)
**Refinement**	
R factor (%)*	18.68
Free R factor (%)*	22.02
RMSD bond lengths (Å)	0.0090
RMSD bond angles (^o^)	1.4472
Ramachadran favoured	296 (100%)
Ramachandran disallowed	0 (0.00%)
PDB accession code	5FII

^*^highest resolution shell shown in parentheses.
