# Supplementary material for: Structural basis for ligand-dependent dimerization of phenylalanine hydroxylase regulatory domain
Source: Sci Rep. 2016 Apr 6;6:23748. doi: 10.1038/srep23748 (PMC4822156; doi:10.1038/srep23748)
Supplement: Supplementary Information [file srep23748-s1.doc]

**Structural basis for ligand-dependent dimerization of phenylalanine hydroxylase regulatory domain**

Dipali Patel^1,2^, Jolanta Kopec^1^, Fiona Fitzpatrick^1,3^, Thomas J. McCorvie^1^, Wyatt W. Yue^1*^

^1^Structural Genomics Consortium, Nuffield Department of Clinical Medicine, University of Oxford, UK OX3 7DQ

^2^ Present address: Department of Biochemistry, University of Oxford, South Parks Road, Oxford, OX1 3QU

^3^Present address: University of Cambridge, MRC Mitochondrial Biology Unit, Wellcome Trust/MRC Building, Hills Road, Cambridge, CB@ OXY

^*^To whom correspondence may be addressed:

W.W.Y. : Tel: +44 (0)1865 617757, Fax:+44 (0)1865 617575, Email: [wyatt.yue@sgc.ox.ac.uk](mailto:wyatt.yue@sgc.ox.ac.uk)

**Supplementary information**

**Supplementary Table 1.** List of hydrogen bonds between subunits at the dimer interface of hPAH-RD.

**Supplementary Fig. S1.** Omit Fo-Fc electron density map of the Phe binding site.

**Supplementary Fig. S2.** Dimerization of other AAAH regulatory domains is not dependent upon binding to their respective amino acid substrate.

|  | [**Monomer 1**](javascript:openWindow('pi_ipage_atom1.html',400,250);) | [**Dist. [Å]**](javascript:openWindow('pi_ipage_atmdist.html',400,250);) | [**Monomer 2**](javascript:openWindow('pi_ipage_atom2.html',400,250);) |
| --- | --- | --- | --- |
|  | GLY  46[ N  ] | 3.73 | PHE 901[ OXT] |
|  | ALA  47[ N  ] | 3.23 | PHE 901[ OXT] |
|  | LEU  48[ N  ] | 3.10 | PHE 901[ OXT] |
|  | ASN  61[ ND2] | 2.77 | GLU  43[ OE2] |
|  | HIS  64[ ND1] | 3.73 | SER  67[ OG ] |
|  | ILE  65[ N  ] | 2.93 | SER  67[ OG ] |
|  | SER  67[ N  ] | 2.95 | ILE  65[ O  ] |
|  | SER  67[ OG ] | 3.58 | ILE  65[ O  ] |
|  |  |  |  |
|  | PHE 901[ O  ] | 3.74 | GLY  46[ N  ] |
|  | PHE 901[ O  ] | 3.19 | ALA  47[ N  ] |
|  | PHE 901[ O  ] | 3.13 | LEU  48[ N  ] |
|  | GLU  43[ OE2] | 2.93 | ASN  61[ ND2] |
|  | SER  67[ OG ] | 3.85 | HIS  64[ ND1] |
|  | SER  67[ OG ] | 2.91 | ILE  65[ N  ] |
|  | ILE  65[ O  ] | 2.90 | SER  67[ N  ] |
|  | ILE  65[ O  ] | 3.49 | SER  67[ OG ] |
|  |  |  |  |

**Supplementary Table 1.** List of hydrogen bonds between subunits at the dimer interface of hPAH-RD. The donors and accepters are listed with residue names, residue numbers and atom labels.


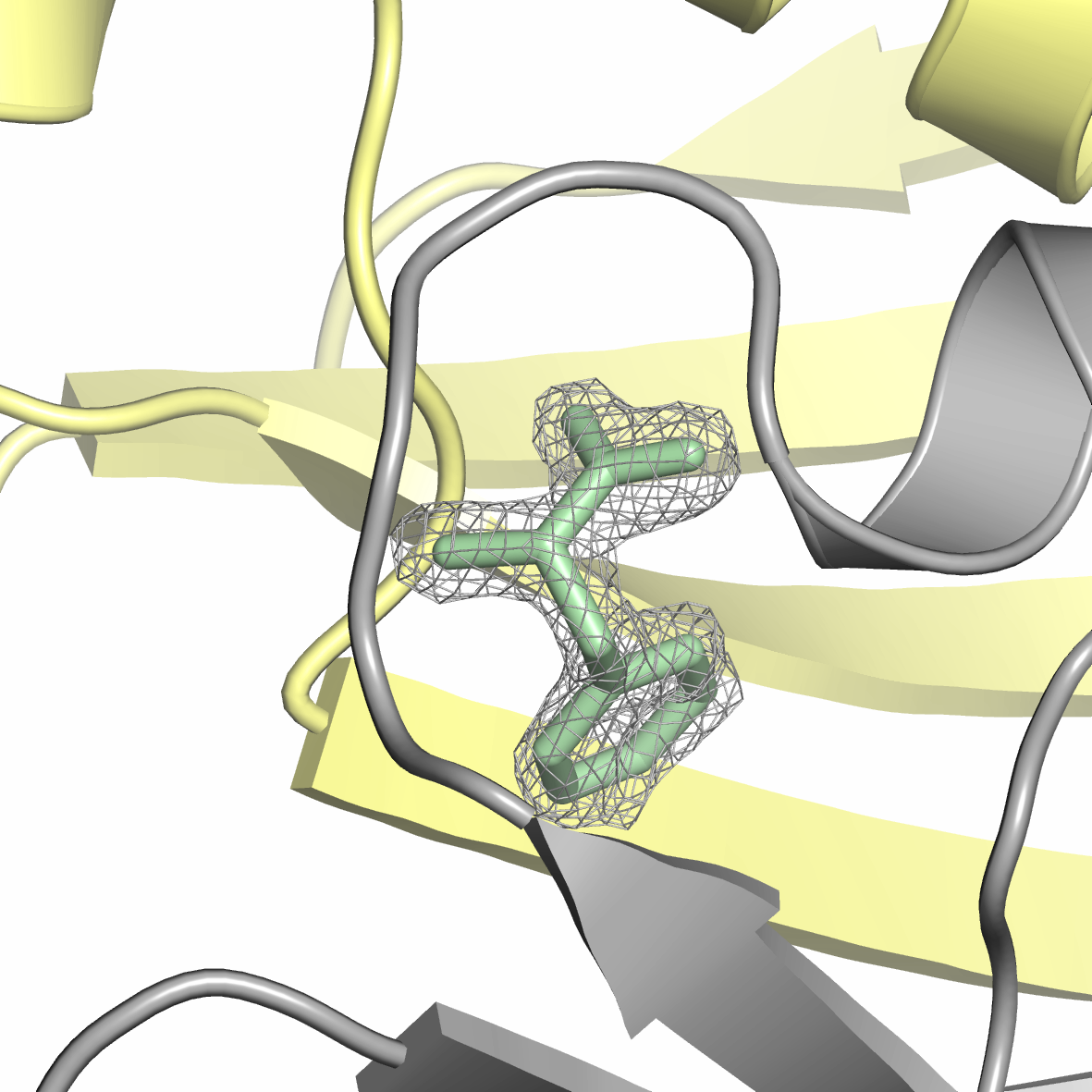


**Supplementary Fig. S1.** Omit Fo-Fc electron density map of the Phe binding site, contoured at 2.5σ.

**Supplementary Fig. S2.** Dimerization of other AAAH regulatory domains is not dependent upon binding to their respective amino acid substrate. (*a*) Schematic of the regulatory domains of human TH-RD and TPH1-RD. (*b*) DSF of the unliganded (grey line; Tm = 59.0°C ± 0.01 SD) and tryptophan-present (pink line; Tm = 58.9°C ± 0.03 SD) TPH1-RD. (*c*) DSF of the unliganded (grey line; Tm = 61.0°C ± 0.1 SD) and tyrosine-bound (pink line) TH-RD. (*d*) SEC-MALS of unliganded (grey line; Tm = 61.8°C ± 0.2 SD) THB-RD.
